# A Rare Foreign Body: An Intracolonic Syringoperitoneal Shunt

**DOI:** 10.14309/crj.0000000000000772

**Published:** 2022-05-04

**Authors:** Nicholas M. McDonald, Elizabeth Aby, Stuart Amateau, Joshua A. Sloan

**Affiliations:** 1 Division of Gastroenterology, Hepatology, and Nutrition, University of Minnesota, Minneapolis, MN

## CASE REPORT

A 55-year-old man with a history of C6 spinal cord injury, paraplegia, and syrinx, requiring a syringoperitoneal shunt 10 years earlier, was admitted to the intensive care unit with sepsis because of sacral osteomyelitis. An abdominal radiograph was obtained suggesting the tip of the syringoperitoneal shunt was within the colon. Subsequent computed tomography of the abdomen confirmed the syringoperitoneal shunt was terminating within the colon (**a**). A colonoscopy was performed, and the shunt, eroding through the wall of the descending colon, was visualized (**b**). After the shunt was disconnected by neurosurgery, a repeat colonoscopy was performed. The mucosa surrounding the shunt was tattooed to help with identification of the defect, followed by retrieval of the shunt with a cold snare. After the shunt was successfully retrieved, an over-the-scope clip (12/6 T, OTSC; Ovesco Endoscopy, Tuebingen, Germany) was deployed with adequate closure of the defect (**c**). Although the defect was apparent with the shunt in place, after removal, the area was difficult to identify with a capped device. In this case, the tattoo proved critical for identification of the defect for closure, and the use of tattoo can be considered for similar cases. Clinically, the patient did well after the procedure without any resultant sepsis and was treated with a 5-day course of oral antibiotics. In similar cases of ventriculoperitoneal shunt causing small intestine or colonic perforation, patients may present with meningitis, peritonitis, or abdominal pain. The standard of care has been surgical resection after disconnection of the catheter.^1–3^ Here, we presented a case showing the feasibility of endoscopic closure in cases of colonic perforation because of a syringoperitoneal shunt.

**Figure 1.**
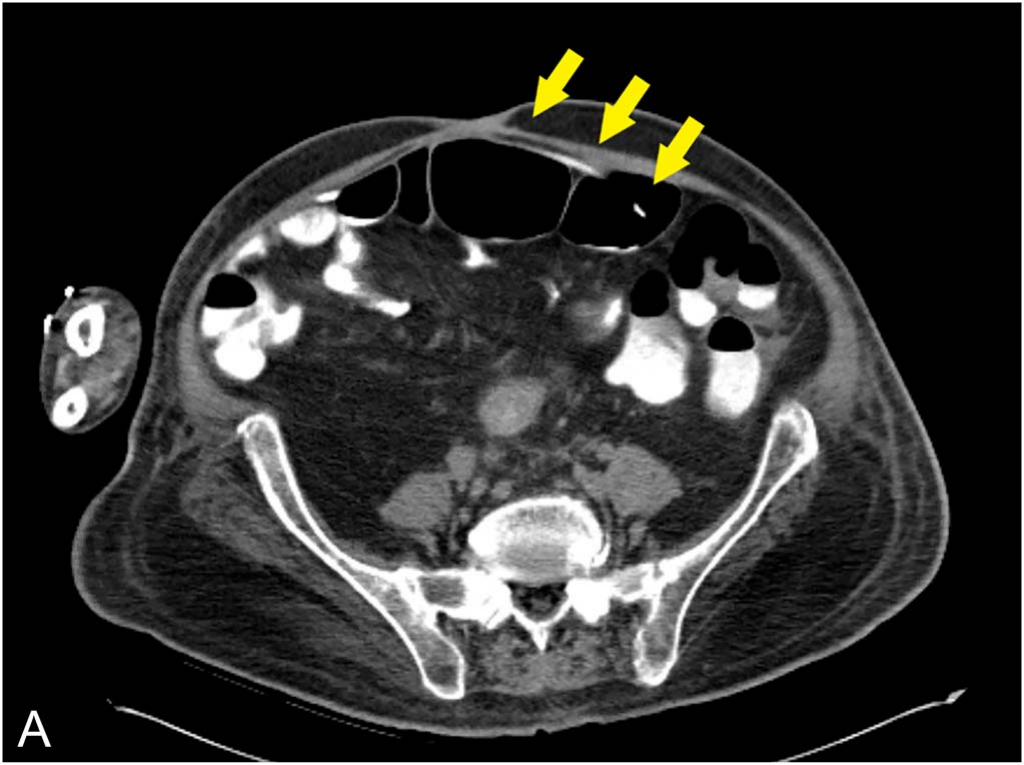
Abdominal computed tomography showing the syringoperitoneal shunt was terminating within the colon.

**Figure 2.**
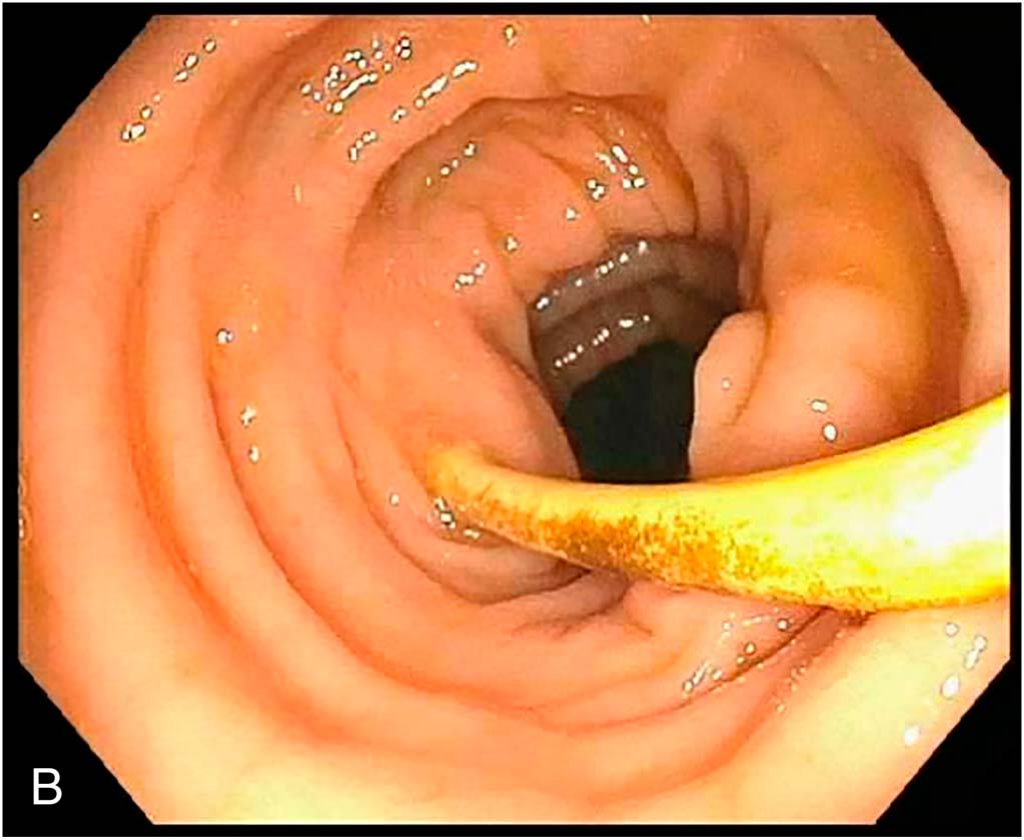
Colonoscopy showing the shunt eroding through the wall of the descending colon.

**Figure 3.**
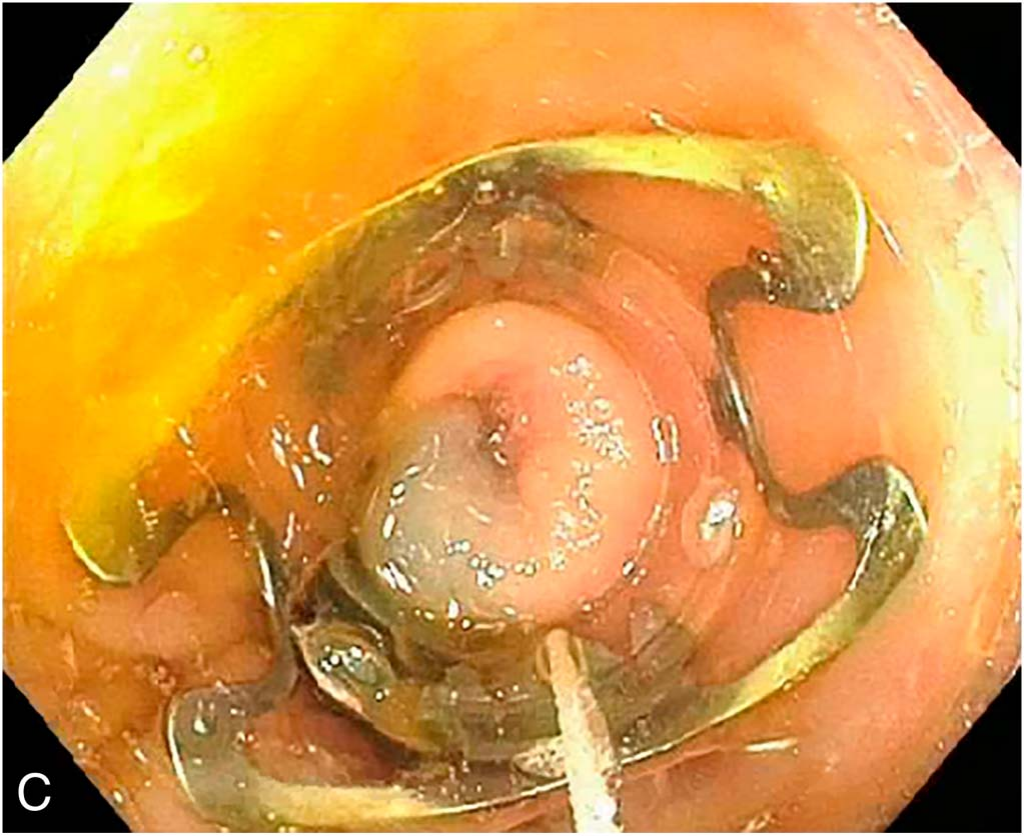
An over-the-scope clip closing the defect.

## DISCLOSURES

Author contributions: NM McDonald wrote the manuscript and is the article guarantor. NM McDonald, JA Sloan, and S. Amateau performed the procedures. All authors performed critical review and editing of the manuscript.

Financial disclosure: None to report.

Informed consent for publication was obtained from the patient. All identifying patient information has been removed to protect patient privacy.
